# Knowledge of obstetric danger signs and associated factors among pregnant women attending antenatal care at health facilities of Yirgacheffe town, Gedeo zone, Southern Ethiopia

**DOI:** 10.1186/s13690-017-0203-y

**Published:** 2017-08-14

**Authors:** Desalegn Tsegaw Hibstu, Yadeshi Demisse Siyoum

**Affiliations:** 1 Department of Reproductive Health, Hawassa University, College of Medicine and Health Sciences, School of Public and Environmental Health, Hawassa, Ethiopia; 2 Department of Public Health, Hawassa University, College of Medicine and Health Sciences, School of Public and Environmental Health, Hawassa, Ethiopia

**Keywords:** Obstetric danger signs, Knowledge, Yirgacheffe, Ethiopia

## Abstract

**Background:**

Obstetric danger signs are not the literal obstetric complications, merely symptoms that are well named by non-clinical personnel. The identification of these danger signs and its relation with complications during pregnancy would increase the capacity of women, their partners and families to seek for timely health care, following the appropriate steps to insure a safe birth and post-partum. The aim of this study was to assess the knowledge of obstetric danger signs and associated factors among pregnant women attending antenatal care in Yirgacheffe town, Gedeo zone, Southern Ethiopia.

**Method:**

Institutional-based cross-sectional study was conducted from March 15–April 15, 2016. Data on pregnant women were collected using a pre-tested and interviewer administered structured questionnaire from 342 women using systematic random sampling technique. Bivariate and multivariate logistic regression was performed using SPSS version 20.0 software.

**Result:**

A total of 342 (90%) pregnant women were included in the study. The level of obstetric knowledge of danger signs was 21.9% (95% CI: 20.2–55.65%). Maternal education (AOR = 0.26, CI: 0.08, 0.88), paternal education (AOR = 0.13, CI; 0.04, 04) and time taken to reach health facilities on foot (AOR = 0.06, CI: 0.02, 0.17) were negatively associated factors while maternal age (AOR = 3.68, CI: 1.30, 10.46), paternal occupation (AOR = 4.65, CI: 1.82, 11.87), place of residence (AOR = 2.61, CI: 1.35, 5.04) were positively associated factors with knowledge of obstetric danger signs.

**Conclusion:**

Maternal and paternal education, maternal age, paternal occupation, place of residence and time taken to reach health facility on foot were the main factors for knowledge of obstetric danger signs. Increasing knowledge of key danger signs, creating and promoting income generating mechanisms need to be continuously done at the health facility and the community as it makes ready women and their families for prompt and appropriate decisions and measures in case of obstetric danger signs.

## Background

Women worldwide get pregnant, and about 10% of them will potentially develop complications that will demand experienced/skilled obstetric care to avert death or life threatening problems [1]. It has also been attested that every day about 14,000 women lose their lives as a result of complications from pregnancy and childbirth [2].

In many developing nations including Ethiopia, maternal mortality yet remains a significant burden and therefore change towards Millennium Development Goal (MDG) five has been particularly disregarded [1]. In developing countries, maternal mortality ratio (MMR) is 15 times higher compared to developed countries. Africa, in particular Sub Saharan countries suffered from the highest MMR at 500 maternal deaths per 100,000 live births [3].

A systematic analysis towards the progress of Millennium Development Goal 5 showed that more than 50% of all maternal deaths in 2008 were in only six countries (India, Nigeria, Pakistan, Afghanistan, Ethiopia, and the Democratic Republic of the Congo) [4]. The magnitude of maternal mortality and morbidity in Ethiopia are among the highest in the globe. Ethiopian demography and health survey (EDHS) reports from 2005 and 2011 showed that MMR was 673 and 676 per 100,000 live births, respectively [5, 6].

The danger signs are not the literal obstetric complications, merely symptoms that are well named by non-clinical personnel. The most frequently occurring danger signs during pregnancy include severe vaginal bleeding, swollen hands/face and blurred vision. The fundamental danger signs during labor and childbirth include severe vaginal bleeding, pro-longed labor, convulsions, and retained placenta. Danger signs during the postpartum period include severe bleeding following childbirth, loss of consciousness after childbirth, and fever [7, 8]. The identification of these danger signs and its relation with complications during pregnancy would increase the capacity of women, their partners and families to seek for timely health care, following the appropriate steps to insure a safe birth and post-partum [9]. One study in Ethiopia by ‘Hailu M., et al. [10] concluded that timely recognition of these danger signs is central to the survival of women.

Approximately 80% of maternal deaths globally occurred due to direct obstetric complications (postpartum severe bleeding, infections following delivery, unsafe induced abortion, and hypertensive disorders in pregnancy and obstructed labour). Hemorrhage solely accounts for one third of all maternal deaths in Africa; still many of these deaths are avoidable. Indirect causes such as malaria, diabetes, hepatitis, anemia and other cardiovascular disorders aggravated by pregnancy can also lead to maternal death [11, 4].

In recent decades, numerous strategies have been carried out for the betterment of maternal health throughout the world [12], the basic aim of antenatal care is to identify the main symptoms and prevent the appearance of life threatening complications during pregnancy, thus addressing the pregnant women’s information needs and providing advice and reassurance [13].

The national reproductive strategy of Ethiopia has given stress to maternal and newborn health to cut down the increased maternal and neonatal mortality. Two of the strategies are empowering women, men, families, and communities to recognize pregnancy-related risks, and to take responsibility for developing and implementing appropriate responses to them and creating an environment supportive to safe motherhood and newborn health [14].

The Federal Ministry of Health (FMOH), reproductive health department and health bureaus of respective regions in Ethiopia have made cooperative effort to promote knowledge of women about obstetric danger signs to decrease maternal mortality to an acceptable level. They have been applying multiple approaches at local and national levels to have better access to health care information across the country including activities as training of health care providers and health extension workers, organizing civil societies, supporting women to women’s associations (such as Health development army, women’s networks, etc.), increasing access to health facilities and allocating health resources equitably among rural and urban areas [15].

Though, big emphasis is given by the national strategy to raise knowledge of obstetric danger signs, there is little information about the current level of knowledge and the determining factors in Ethiopia [10]. Therefore, this study was carried out to determine the level of knowledge about obstetric danger signs and associated factors among pregnant women attending antenatal care in Yirgacheffe town, Gedeo zone, Southern Nation, Nationalities and People Region (SNNPR), Ethiopia.

## Methods

### Study setting and period

The study was conducted in Yirgacheffe town, Gedeo zone, from March 15–April 15, 2016, which is known for producing coffee as an important cash crop globally. It is located in SNNPR, Ethiopia at a distance of 395 km from Addis Ababa, capital city of Ethiopia and 125 km from Hawassa, capital city of SNNPR.

The town has two health centers providing preventive, promotive, and curative and rehabilitation services for the community of the town and the surrounding community. The health center provides health education on regular basis for women attending antenatal visits on danger signs that occur during pregnancy, child birth and postpartum period. It also provides different laboratory tests including hemoglobin, urinalysis, sexually transmitted diseases and appropriate physical examination during their antenatal visits.

## Study design and populations

An institutional-based quantitative cross-sectional study was employed. The source population for this study was all pregnant women found in Yirgacheffe town and the study populations were pregnant women attending ante natal care at health facilities of the town.

### Sample size and sampling procedure

Sample size was determined using single population proportion formula by considering the following assumption: 95% confidence level, 5% margin of error and proportion of knowledge about obstetric danger signs 45.9% [10].

$$ n=\frac{{\left( z\alpha / 2\right)}^2 p\left( 1\hbox{-} p\right)}{} =\frac{{\left( 1. 96\right)}^2\times 0.459(0.541)}{d^2}=\frac{3.84\times 0.248}{(0.05)^2}= 381 $$

Study participants were selected by systematic random sampling technique with an interval of three where the interval constant was obtained by dividing the total ANC attendants to the sample size from their antenatal care registration book.

### Operational definitions

*Knowledge of women about obstetric danger signs* were measured by the total number of correct spontaneous answers to 9 items on knowledge of pregnancy danger signs and 7 items on knowledge of labor and childbirth danger signs and 7 items on knowledge of postpartum danger signs with a minimum score of 0 and maximum of 9.

Accordingly, three categories were developed for pregnancy, labor/childbirth and post-partum*. Knowledgeable on key danger signs of pregnancy*: a woman was considered as knowledgeable when she mentioned at least three key danger signs for pregnancy spontaneously. *Knowledgeable on key danger signs of labor/childbirth*: a woman was considered as knowledgeable when she mentioned at least three key danger signs for labor/childbirth spontaneously. *Knowledgeable on key danger signs of post-partum*: a woman was considered knowledgeable if when she mentioned at least the three key danger signs for post-partum spontaneously [16]. The dependent variable was later dichotomized as *knowledgeable* and *not-knowledgeable*.

### Data collection tool and procedure

Pre-tested and interviewer-administered structured questionnaires were used. Fifth year female medical students were recruited to collect the data. The questionnaire was prepared in English language and translated to Amharic (local language) and translated back to English to maintain the consistence of the data. Training was given to data collectors for two days. Two master’s degree holders supervised the overall data collection and check for filled questionnaire for consistency and completeness. During data collection, questionnaires were reviewed and checked for completeness and relevance by the principal investigators and the supervisors.

### Data processing and analysis

After data collection, data were edited and cleaned before data analysis, each questionnaire was checked for completeness and code was given before data entry. Data was cleaned and entered into computer by using EPI Info version 3.5.3 and the analysis was done using SPSS version 20.0.

Frequency, percentage and descriptive summaries were used to describe the study variable using univarite analysis. Logistic regression was carried out to identify factors associated with knowledge of obstetric danger signs. Variables with *p*-value ≤0.2 in the bivariate analysis were selected as candidate variables for multivariate logistic regression analysis to control the effect of confounders. Adjusted odds ratios with their 95% confidence intervals and *p*-value of less than 0.05 were considered to have significant association between the outcome and the explanatory variables. The model fitness was checked using Hosmer and Lemeshow.

## Results

### Socio demographic and economic characteristics

A total of 342 pregnant women were included in the study with a response rate of 90%. The minimum and the maximum age of the respondents were 16 years and 43 years, respectively. The mean age of the study subjects was 26.2 years (±6.2, standard deviation (SD)). One hundred nineteen (34.8%) study subjects were found in the age group 15–24 years.

Almost half 174 (50.9%) of the respondents were protestant by religion followed by orthodox 106 (31%), and 198 (57.9%) were Gedeo by ethnicity. Of the total study participants 333 (97.4%) were married with average family size of 4.23 (±1.846 SD). Nearly three among four study subjects, 252 (73.7%) decided health service utilization by themselves and 52 (15.2%) were leaders in the family (Table 1).

**Table 1:** Socio demographic, socio economic and reproductive history characteristics of the study subjects, Yirgacheffe town, Gedeo zone, Southern Ethiopia, 2016

Variables	Frequency	Percentage
Age		
15–24	119	34.8
25–34	166	48.5
> =35	57	16.7
Religion		
Protestant	174	50.9
Orthodox	106	31.0
Catholic	20	5.8
Muslim	42	12.3
Ethnicity		
Gedeo	198	57.9
Guraghe	55	16.1
Siliti	29	8.5
Kembata	27	7.9
Sidama	24	7.0
Wolaita	9	2.6
Marital status		
Single	9	2.6
Married	333	97.4
Family size		
< =4	212	62.0
> 4	130	38.0
Role in the family		
Leaders	52	15.2
Members	290	84.8
Decision maker about service utilization		
Self	252	73.7
Other person	90	26.3
Maternal education		
Unable to read and write	109	31.9
Read and write(No formal education)	84	24.6
Primary education	80	23.4
Secondary and above	69	20.2
Parental education		
Unable to read and write	37	10.8
Read and write(No formal education)	49	14.3
Primary education	129	37.7
Secondary and above	127	37.1
Maternal occupation		
Housewife	231	67.5
Governmental employed	64	18.7
Merchant	47	13.8
Paternal Occupation		
Governmental employed	87	25.4
Farmer	96	28.1
Merchant	145	42.4
Daily laborer	14	4.1
Monthly Income		
< 1000 birr	104	30.4
1001–2000 birr	107	31.3
2001–3000 birr	58	17.0
> 30,000 birr	73	21.3
Place of Residence		
Urban	203	59.4
Rural	139	40.6
Duration of pregnancy		
First trimester	17	5.0
Second trimester	134	39.2
Third trimester	191	55.8
Age at first pregnancy		
15–24 years	326	95.3
> 24 years	16	4.7
Number of pregnancy		
1	85	24.9
> =2	257	75.1
Number of parity		
1–4	232	67.8
> 4	110	32.2
Number of ANC follow up		
1	109	31.9
2	82	24.0
3	84	24.6
4	67	19.5
Time taken to health facility(on foot)		
< 30 min	309	90.4
≥ 30 min	33	9.6

Concerning education status, one among five, 69 (20.2%) women attended secondary and above school, and 127 (37.1%) of their partners/husbands attended secondary and above school. Almost seven among ten pregnant women (67.5%) were house wife by occupation and 87 (25.4%) of their counterpart were governmental employed. One hundred four study participants (30.4%) were living in rural areas (Table 1).

### Reproductive history

Three hundred twenty six (95.3%) of the study participants were in the age group 15–24 years old during their first pregnancy. Three among four, 257 (75.1%) had history of two or above pregnancy. Three among five 203 (59.4%) study participants delivered their last child in a health facility. The majority, 309 (90.4%) of participants reported that below thirty minute is required to reach the health institution. One hundred nine (31.9%) and 67 (19.5%) of the study participants had their first and fourth ANC visits during their pregnancy period, respectively (Table 1).

### Knowledge of obstetric danger signs

The overall knowledge of study participants regarding obstetric danger signs was 21.9% (95% CI: 20.2–55.65%). Almost half of the study subjects, 168 (49.1%) were found to be knowledgeable about danger signs during pregnancy. It was also found that 181 (52.9%) participants were knowledgeable about danger signs during child birth. One hundred fifty three, 153 (44.7%) study subjects were knowledgeable about danger signs during postpartum period. Severe vaginal bleeding was the most frequently known danger sign mentioned by study participants during pregnancy 258 (75.4%), 252 (73.7%), labor and child birth, and 158 (46.2%) at postpartum period (Table 2).

**Table 2:** Knowledge of obstetric danger signs during pregnancy and post-partum period in Yirgacheffe town, Gedeo zone, Southern Ethiopia, 2016

Characteristics	Knowledge of danger signs during:					
	Pregnancy		Labor and child birth		Post-partum	
	Frequency	Percentage	Frequency	Percentage	Frequency	Percentage
Vaginal bleeding						
No	84	24.6	90	26.3	184	53.8
Yes	258	75.4	252	73.7	158	46.2
Difficulty of breathing						
No	317	92.7	NA	NA	261	76.3
Yes	25	7.3	NA	NA	81	23.7
Loss of consciousness						
No	314	91.8	317	92.7	280	81.9
yes	28	8.2	25	7.3	62	18.1
High fever						
No	298	87.1	272	79.5	NA	NA
Yes	44	12.9	70	20.5	NA	NA
Severe Headache						
No	237	69.3	261	76.3	265	77.5
Yes	105	30.7	81	23.7	77	22.5
Severe abdominal pain						
No	182	53.2	NA	NA	NA	NA
Yes	160	46.8	NA	NA	NA	NA
Blurred vision						
No	224	65.5	NA	NA	255	74.6
Yes	118	34.5	NA	NA	87	25.4
Convulsion						
No	329	96.2	293	85.7	332	97.1
Yes	13	3.8	49	14.3	10	2.9
Swollen Hand/face						
No	279	81.6	NA	NA	293	85.7
Yes	63	18.4	NA	NA	49	14.3
Vaginal discharge						
No	NA	NA	NA	NA	281	82.2
Yes	NA	NA	NA	NA	61	17.8
Mal-position/presentation						
No	NA	NA	235	68.7	NA	NA
Yes	NA	NA	107	31.3	NA	NA
Retained placenta						
No	NA	NA	230	67.3	NA	NA
Yes	NA	NA	112	32.7	NA	NA
Labor lasting >12 h						
No	NA	NA	194	56.7	NA	NA
Yes	NA	NA	148	43.3	NA	NA

*NA* indicates that the specific danger sign was not asked

### Associated factors with knowledge of obstetric danger signs

In the multivariate analysis maternal age, maternal and paternal education, paternal occupation, time taken on foot to the health facility and place of residence were significantly associated with knowledge of obstetric danger signs.

The study demonstrated that women in the age group 25–34 years old were 3.5 (AOR = 3.68, CI: 1.30, 10.46) times more likely to be knowledgeable of obstetric danger signs in relation with women who were 35 years old and above.

Women who can read and write but without formal education was 75% (AOR = 0.26, CI: 0. 08, 0.88) less likely to be knowledgeable about obstetric danger signs compared to women who attended secondary and above education.

It was also observed that the probability of having knowledge of obstetric danger signs decreased almost by 90% (AOR = 0.13, CI; 0.04, 0.40) among women whose partners/husbands did not read and write, than those whose literacy status was secondary and above education.

Another important predictor was parental/husband occupation and place of residence. The chance of having knowledge of obstetric danger signs was 5 times (AOR = 4.65, CI: 1.82, 11.87) higher among women whose husbands were merchant by occupation compared with governmental employed. Women living in urban areas were 3 times (AOR = 2.61, CI: 1.35, 5.04) more likely to have knowledge of obstetric danger signs compared to those living in rural areas.

On the other hand, knowledge of obstetric danger signs was 95% (AOR = 0. 06, CI: 0.02, 0.17) less likely among women who travelled on foot more than 30 min to health facilities for health service utilization (Table 3).

**Table 3:** Factors associated with knowledge of obstetric danger signs in Yirgacheffe town, Gedeo zone, Southern Ethiopia, 2016

Variables	Knowledge of obstetric danger signs		COR (95% CI)	AOR (95% CI)	*P*-value
	Not knowledgeable	Knowledgeable			
Maternal age					
15–24	102	17	1.02 (0.41, 2.53)	1.33 (0.42, 4.17)	0.230
25–34	116	50	2.64 (1.17, 5.98)	3.68 (1.30, 10.46)	0.001
> =35	49	8	1		
Maternal education					
Unable to read and write	89	20	0.48 (0.24,0. 97)	0.33 (0.10, 1.07)	0.433
Read and write(no formal education)	74	10	0.29 (0.13, 0.66)	0.26 (0. 08, 0.88)	0.010
Primary education	57	23	0.86 (0.43, 1.74)	0.79 (0.30, 2.09)	0.445
Secondary and above	47	22	1	1	
Paternal education					
Unable to read and write	30	7	0.46 (0.19, 1.12)	0.13 (0.04, 0.40)	0.006
Read and write(no formal education)	41	8	0.38 (0.16–0.89)	0.08 (0.02, 0.24)	0.045
Primary education	112	17	0.30 (0.16–0.56)	0.10 (0.04, 0.25)	0.005
Secondary and above	84	43	1	1	
Paternal occupation					
Governmental employed	71	16	1	1	
Farmer	75	21	1.24 (0.60, 2.57)	0.05 (0.01, 0.26)	0.016
Merchant	113	32	1.26 (1.64, 2.46)	4.65 (1.82, 11.87)	0.001
Daily laborer	8	6	3.33 (0.02, 10.9)	0.28 (0.06, 1.32)	0.245
Place of residence					
Urban	170	33	2.23 (1.33, 3.75)	2.61 (1.35, 5.04)	0.000
Rural	97	42	1	1	
Distance to Health facility on foot					
< 30 min	12	21	1	1	
≥ 30	255	54	0.12 (0.06, 0.26)	0. 06 (0.02, 0.17)	0.000

Hosmer and Lemeshow for model fitness is 0.876

## Discussion

Every minute, a woman dies due to causes related to pregnancy, childbirth and postnatal period. These causes are avoidable if women with complications are able to identify and seek appropriate emergency obstetric care, which makes a difference between life and death. Thus, this study had tried to determine the level of knowledge about obstetric danger signs and associated factors among pregnant women attending antenatal care in Yirgacheffe town, Gedeo zone, Southern Nation, Nationalities and People Region, Ethiopia.

In this study, it was revealed that the overall knowledge of obstetric danger signs was 21.9%. This finding is congruent with the studies conducted in Ethiopia: Alettawondo (30.9%) and Arba Minch (24.1%), SNNPR [10, 16]; Debre-Birhan (38.6%), Amhara Region [17].

On the other hand, in this study about 75% of the study subjects explained that vaginal bleeding as danger sign during pregnancy which was higher than the findings in Burkina Faso (39.4%) [18], Guatemala (31.0%) [19] and Tigray, Ethiopia (49.1%) [20]. This variation might be due to socio-cultural difference and difference in implementation of relevant health intervention programs such as provision of antenatal care and delivery services. The finding of the study also indicated that half, 168 (49.1%) of the respondents were knowledgeable about the danger signs of pregnancy which is inconsistent with findings in Tigray, Ethiopia and India [20, 21], respectively.

Significant association was observed concerning maternal age. It was figured out that knowledge of obstetric danger signs was more likely to increase among women with the age group 25–34 years old. This finding is congruent with studies conducted in Ethiopia, Tanzania, Enugu State, Nigeria and South East Nigeria, [16, 22–24], respectively. It might be explained as elder women not only own better knowledge of obstetric danger signs in this age group but also they are psychologically and physically ready to accept information on danger signs.

Maternal and paternal/husband education were identified a predictor for knowledge of obstetric danger signs. It was indicated that the level of obstetric danger signs knowledge was increased among women and husbands attended secondary and above education compared with those with no education. This study is in line with other studies [16, 20, 23, 25–27]. This might be explained by the fact that there is no question that educated women can have better information and care for themselves. Education provides better health knowledge, improves the effectiveness of health behavior and enables to take prompt measures when the danger signs arise.

The place of residence was also found as a determinant of knowledge of obstetric danger signs. The study found that obstetric danger signs knowledge of women were more likely to increase among women living in urban residence in comparison with rural areas. This finding was supported by the findings in Ethiopia: Erer district, Somali region and Goba district region [26–28] and a study in Southeast Nigeria [24]. This is probably because urban areas are exposed to different health care services including higher coverage with health information dissemination.

On the other hand, probably because better access to health facility and health information due to increased access to health facilities, it was pointed out that knowledge of obstetric danger signs was more likely to increase among women who travelled less than thirty minutes on foot for health service utilization. This study was in agreement with the findings in other areas [24, 27].

The paternal/husband occupational status in this study was significantly associated with maternal knowledge of obstetric danger signs. The probability of obstetric danger signs knowledge was highest among women whose husbands were merchants by occupation. This study is agreed with the finding in Nigeria [24]. This might happen because merchant fathers would have a positive outcome in knowledge of obstetric danger signs by increasing household income and economic status of the family and as a result women might get frequent health institution contact for antenatal care, delivery and health information about obstetric danger signs.

While interpreting the findings of this study, scholars need to take into consideration the following limitations. First, the cross sectional nature of the data had made impossible to arrive at the causal relation between the different explanatory variables and knowledge of women about obstetric danger signs. Second, an expected proportion of knowledge about obstetric danger signs of the women in the study (*N* = 381) was not reached, as only 342 pregnant women were included with a response rate of 90%. Third, as the study is based on a sample of all pregnant women attending antenatal care, possible selection bias needs to be considered since it did not address the main characteristic of the general population in the study.

## Conclusions

In general, in this study, the level of obstetric danger signs knowledge was low. Maternal and husband education, paternal occupational, maternal age, place of residence and time taken to reach health facility on foot were identified as the significant factors for knowledge of obstetric danger signs among pregnant women in Yirgacheffe town, Gedeo zone, Southern Ethiopia.

Increasing knowledge or awareness of key obstetric danger signs need to be given focus as it makes women and their families ready for prompt and appropriate decisions and measures in case of obstetric danger signs among pregnant women. Health education dissemination strategies on obstetric danger signs and creating and promoting income generating mechanisms need to be continuously done at the health facility and community.

## Abbreviations

ANC: Ante natal care
AOR: Adjusted odd ratio
CI: Confidence interval
COR: Crude odd ratio
EDHS: Ethiopian demography and health survey
FMOH: Federal Ministry of Health
MDG: Millennium development goal
MMR: Maternal mortality ratio
SD: Standard deviation
SNNPR: Southern Nation Nationalities and People Region
SPSS: Statistical package for social science
UN: United Nations
WHO: World Health Organization
